# Supplementary material for: An open-source framework for large-scale, flexible evaluation of biomedical text mining systems
Source: J Biomed Discov Collab. 2008 Jan 29;3:1. doi: 10.1186/1747-5333-3-1 (PMC2276192; doi:10.1186/1747-5333-3-1)
Supplement: Additional file 1 — Evaluation platform source code. The additional file contains the software evaluation framework discussed in this paper. The most current release can be downloaded from SourceForge.net [1]. See the accompanying README file for instructions on installation and use. Also included in the distribution is a collection of UIMA wrappers for some commonly used BioNLP tools and annotated corpora. [file 1747-5333-3-1-S1.zip › bionlp_uima_repository-1.0.1/docs/api/edu/uchsc/ccp/uima/cc/summary/comparison/ComparatorConfigurator.html]

ComparatorConfigurator (Center for Computational Pharmacology Util/UIMA API)


|  |  |  |
| --- | --- | --- |
| **Overview** \| **Package** \| **Class** \| **Tree** \| **Deprecated** \| **Index** \| **Help** | |  |
| **PREV CLASS**   **NEXT CLASS** | **FRAMES**    **NO FRAMES**     **All Classes** |  |
| SUMMARY: NESTED \| FIELD \| CONSTR \| METHOD | DETAIL: FIELD \| CONSTR \| METHOD |  |


---


## edu.uchsc.ccp.uima.cc.summary.comparison Class ComparatorConfigurator

```
java.lang.Object
  edu.uchsc.ccp.uima.cc.summary.comparison.ComparatorConfigurator
```

---

``` public class ComparatorConfigurator extends Object ```

This is a utility class for configuring an AnnotationComparator. Its main purpose is to read the config file for the
AnnotationComparator.
The format for the config file is as follows:

```
    <!-- Settings for a single AnnotationComparator run -->
    <AnnotationComparatorSettings>
   
    <!-- AnnotationGroups define the annotations that will be used in the comparison. Each group          -->
    <!-- is identified by a unique GroupID, and has annotation members defined by their                   -->
    <!-- AnnotationSetID, AnnotatorID, and AnnotationType (which is equivalent to the ClassMention type). -->
    <!-- Multiple AnnotationGroups are permitted. -->
    <AnnotationGroup>
       <GroupID>[Group ID]</GroupID>
       <AnnotationSetID>[Annotation Set ID]</AnnotationSetID>
       <AnnotatorID>[Annotator ID]</AnnotatorID>
       <!-- Multiple AnnotationTypes are permitted -->
       <AnnotationType>[Type String]</AnnotationType>
    </AnnotationGroup>
    
    <!?ComparisonGroups define how the annotations are compared. Currently, only pairwise comparisons are -->
    <!-- enabled. The ComparisonGroup with GoldStandard set to true is used as the gold standard, and all -->
    <!-- other groups are compared against it.                                                            -->
    <ComparisonGroup>
       <!-- If the GoldStandard tag is not present, its value is presumed to be false. -->
       <GoldStandard>[true or false]</GoldStandard>
       <ComparisonGroupDescription>[Description String]</ComparisonGroupDescription>
       <!-- Multiple AnnotationGrouPIDs are permitted -->
       <AnnotationGroupID>[Annotation Group ID]</AnnotationGroupID>
    </ComparisonGroup>
    
    </AnnotationComparatorSettings>
```

**Author:**
:   williamb

---

**Constructor Summary**

`ComparatorConfigurator(String configFile)`


| **Method Summary** | |
| --- | --- |
| `Map<AnnotationGroupID,AnnotationGroup>` | `getAnnotationGroupMap()` |
| `List<ComparisonGroup>` | `getComparisonGroupList()` |

**Methods inherited from class java.lang.Object**

`clone, equals, finalize, getClass, hashCode, notify, notifyAll, toString, wait, wait, wait`

**Constructor Detail**

### ComparatorConfigurator

```
public ComparatorConfigurator(String configFile)
```


**Method Detail**

### getAnnotationGroupMap

```
public Map<AnnotationGroupID,AnnotationGroup> getAnnotationGroupMap()
```

---


### getComparisonGroupList

```
public List<ComparisonGroup> getComparisonGroupList()
```


---


|  |  |  |
| --- | --- | --- |
| **Overview** \| **Package** \| **Class** \| **Tree** \| **Deprecated** \| **Index** \| **Help** | |  |
| **PREV CLASS**   **NEXT CLASS** | **FRAMES**    **NO FRAMES**     **All Classes** |  |
| SUMMARY: NESTED \| FIELD \| CONSTR \| METHOD | DETAIL: FIELD \| CONSTR \| METHOD |  |


---
